# Brain structure and function: a multidisciplinary pipeline to study hominoid brain evolution

**DOI:** 10.3389/fnint.2023.1299087

**Published:** 2024-01-08

**Authors:** Angela D. Friederici, Roman M. Wittig, Alfred Anwander, Cornelius Eichner, Tobias Gräßle, Carsten Jäger, Evgeniya Kirilina, Ilona Lipp, Ariane Düx, Luke J. Edwards, Cédric Girard-Buttoz, Anna Jauch, Kathrin S. Kopp, Michael Paquette, Kerrin J. Pine, Steve Unwin, Daniel B. M. Haun, Fabian H. Leendertz, Richard McElreath, Markus Morawski, Philipp Gunz, Nikolaus Weiskopf, Catherine Crockford, EBC Consortium, Daniel Ashoff, Karoline Albig, Bala Amarasekaran, Sam Angedakin, Alfred Anwander, Caroline Asiimwe, Christian Bock, Birgit Blazey, Andreas Bernhard, Jacinta C Beehner, Laurent Bailanda, Raphael Belais, Thore J Bergman, Denny Böttcher, Tatiana Bortolato, Penelope Carlier, Julian Chantrey, Catherine Crockford, Daniela Denk, Tobias Deschner, Ariane Düx, Luke J. Edwards, Cornelius Eichner, Dag Encke, Gelardine Escoubas, Malak Ettaj, Pawel Fedurek, Karina Flores, Alejandra Romero Florero, Richard Franke, Angela D Friederici, Cedric Girard-Buttoz, Jorge Gomez Fortun, Tobias Gräßle, Eva Gruber-Dujardin, Philipp Gunz, Susan Hambrecht, Florian Hansmann, Jess Hartel, Daniel BM Haun, Michael Henshall, Catherine Hobaiter, Noémie Hofman, Jennifer E Jaffe, Carsten Jäger, Anna Jauch, Stomy Karhemere, Evgenya Kirilina, Robert Klopfleisch, Tobias Knauf-Witzens, Kathrin Kopp, Bastian Lange, Kevin E Langergraber, Arne Lawrenz, Kevin Lee, Fabian H Leendertz, Illona Lipp, Matyas Liptovszky, Christelle Patricia Lumbu, Patrice Makouloutou Nzassi, Guy Landry Mamboundou Kouima, Kerstin Mätz-Rensing, Richard McElreath, Zoltan Mezö, Fanny Minesi, Sophie Moittie, Torsten Møller, Markus Morawski, Dave Morgan, Mathias Müller, Timothy Mugabe, Martin Muller, Karin Olofsson-Sannö, Alain Ondzie, Emily Otali, Michael Paquette, Simone Pika, Kerrin J. Pine, Andrea Pizarro, Kamilla Pleh, Sandra Reichler-Danielowski, Jessica Rendel, Martha M Robbins, Konstantin Ruske, Liran Samuni, Crickette Sanz, Jan Schinköthe, André Schüle, Ingo Schwabe, Katarina Schwalm, Anistan Sebastiampillai, Lara Southern, Sheri Speede, Jonas Steiner, Mark F Stidworthy, Martin Surbeck, Claudia A. Szentiks, Tanguy Tanga, Tobias Loubser Theron, Reiner Ulrich, Steve Unwin, Erica van de Waal, Sue Walker, Nikolaus Weiskopf, Gudrun Wibbelt, Navena Widulin, Hermann Will, Roman M Wittig, Kim Wood, Emiliano Zaccarella, Klaus Zuberbühler

**Affiliations:** ^1^Department of Neuropsychology, Max Planck Institute for Human Cognitive and Brain Sciences, Leipzig, Germany; ^2^Evolution of Brain Connectivity Project, Max Planck Institute for Evolutionary Anthropology, Leipzig, Germany; ^3^Institute for Cognitive Sciences Marc Jeannerod, UMR CNRS, University Claude Bernard Lyon, Bron, France; ^4^Taï Chimpanzee Project, CSRS, Abidjan, Côte d'Ivoire; ^5^Epidemiology of Highly Pathogenic Microorganisms, Robert Koch Institute, Berlin, Germany; ^6^Department of Neurophysics, Max Planck Institute for Human Cognitive and Brain Sciences, Leipzig, Germany; ^7^Medical Faculty, Center of Neuropathology and Brain Research, Paul Flechsig Institute, University of Leipzig, Leipzig, Germany; ^8^Helmholtz Institute for One Health, University of Greifswald, Greifswald, Germany; ^9^Department of Comparative Cultural Psychology, Max Planck Institute for Evolutionary Anthropology, Leipzig, Germany; ^10^School of Bioscience, University of Birmingham, Birmingham, United Kingdom; ^11^Department of Human Behavior, Ecology and Culture, Max Planck Institute for Evolutionary Anthropology, Leipzig, Germany; ^12^Department of Human Origins, Max Planck Institute for Evolutionary Anthropology, Leipzig, Germany; ^13^Faculty of Physics and Earth System Sciences, Felix Bloch Institute for Solid State Physics, Leipzig University, Leipzig, Germany

**Keywords:** non-human primates, behavior, structural MRI, histology, hominoid fossil

## Abstract

To decipher the evolution of the hominoid brain and its functions, it is essential to conduct comparative studies in primates, including our closest living relatives. However, strong ethical concerns preclude *in vivo* neuroimaging of great apes. We propose a responsible and multidisciplinary alternative approach that links behavior to brain anatomy in non-human primates from diverse ecological backgrounds. The brains of primates observed in the wild or in captivity are extracted and fixed shortly after natural death, and then studied using advanced MRI neuroimaging and histology to reveal macro- and microstructures. By linking detailed neuroanatomy with observed behavior within and across primate species, our approach provides new perspectives on brain evolution. Combined with endocranial brain imprints extracted from computed tomographic scans of the skulls these data provide a framework for decoding evolutionary changes in hominin fossils. This approach is poised to become a key resource for investigating the evolution and functional differentiation of hominoid brains.

## Introduction

A striking feature of the human species is our large brain (Buckner and Krienen, 2013), and the cognitive ability to use language (Friederici, 2009). Non-human primates also demonstrate complex skills such as extensive tool use (Johnson-Frey, 2004), and sophisticated social cognition. The question of how the neural networks supporting these cognitive and social skills evolved remains unanswered, mainly due to a lack of studies directly comparing human and different non-human primate (NHP) brains and the related behavior. While human brains are frequently compared to those of monkeys like macaques, thorough comparative analyses ideally also encompass information from our nearest living relatives, the great apes, especially chimpanzees and bonobos. Genetic data suggest that living humans and chimpanzees last shared a common ancestor more than 6 million years ago (Langergraber et al., 2012). Consequently, today’s chimpanzees do not reflect the ancestral morphology, but several million years of independent evolution. Nonetheless, a number of macro- and micro-structural brain characteristics, like the expansion of the frontal lobe, the parietal lobe and parts of the temporal lobe and differentiation of cortical myeloarchitecture place great ape brains closer to human brains than to those of species in other primate clades. This suggests that great ape brains show a number of evolutionary derivations since the split with old world monkeys some 20 million years ago. Additionally, the fossil record suggests, that early hominins such as *Australopithecus afarensis* (of “Lucy” fame) had great ape-like brain volumes and brain organization (Gunz et al., 2020). Therefore, analyzing the brains of our closest living relatives, chimpanzees and bonobos, means to look at the results of the evolutionary process and can help shed light on the evolutionary trajectory of the human brain (Rilling, 2014).

*In vivo* methods, such as invasive electrophysiological recordings or neuroimaging of sedated or anaesthetized NHP in human care, have provided some insights into neural networks and brain structures of non-human primates in the past (Van Essen and Glasser, 2018). These methods, however, are invasive and have been banned for use in our closest living relatives, the great apes, in many countries (e.g., US, EU-member states, UK, Japan, New Zealand) (Hutson, 2010; Kaiser, 2013). Thus, alternative methods are needed to gain more insights into the brain structure of great apes. Recent advances now place *post mortem* neuroimaging of extracted brains after natural death as a powerful, high-quality, ethical alternative for investigating brain structures.

A key to uncovering mechanisms of hominid brain evolution is understanding the outstanding plasticity of primate brains (Sherwood and Gómez-Robles, 2017; Froudist-Walsh et al., 2018). Environmental factors shape the brain and the behavior, resulting in environmental variation across individuals and populations (Logan et al., 2018). Understanding brain evolution, therefore, requires examining brains from individuals that have lived in variable socio-ecological environments, importantly, including the species’ natural habitat (Bruner, 2019). Studies conducted only on animals from similar socio-ecological environments, for example from research centers, may limit and systematically bias our knowledge on great ape neuroanatomy and brain plasticity.

For a comprehensive comparison of behaviors and brain structures across primates in different habitats, an interdisciplinary approach is needed, bridging behavioral ecology, anthropology, neurobiology, psychology, physics and other fields. Here we present such a highly interdisciplinary approach with a working pipeline allowing us to collect *in vivo* data on relevant primate behavior, such as vocal communication and tool use, high-quality sampling of their *post mortem* brains, cutting edge neuroimaging and histology as well as computed tomography of endocasts. This pipeline provides a methodological approach to relate behavior and brain structure in our closest relatives, but can certainly also be applied to other species.

### The approach

**Figure 1:**
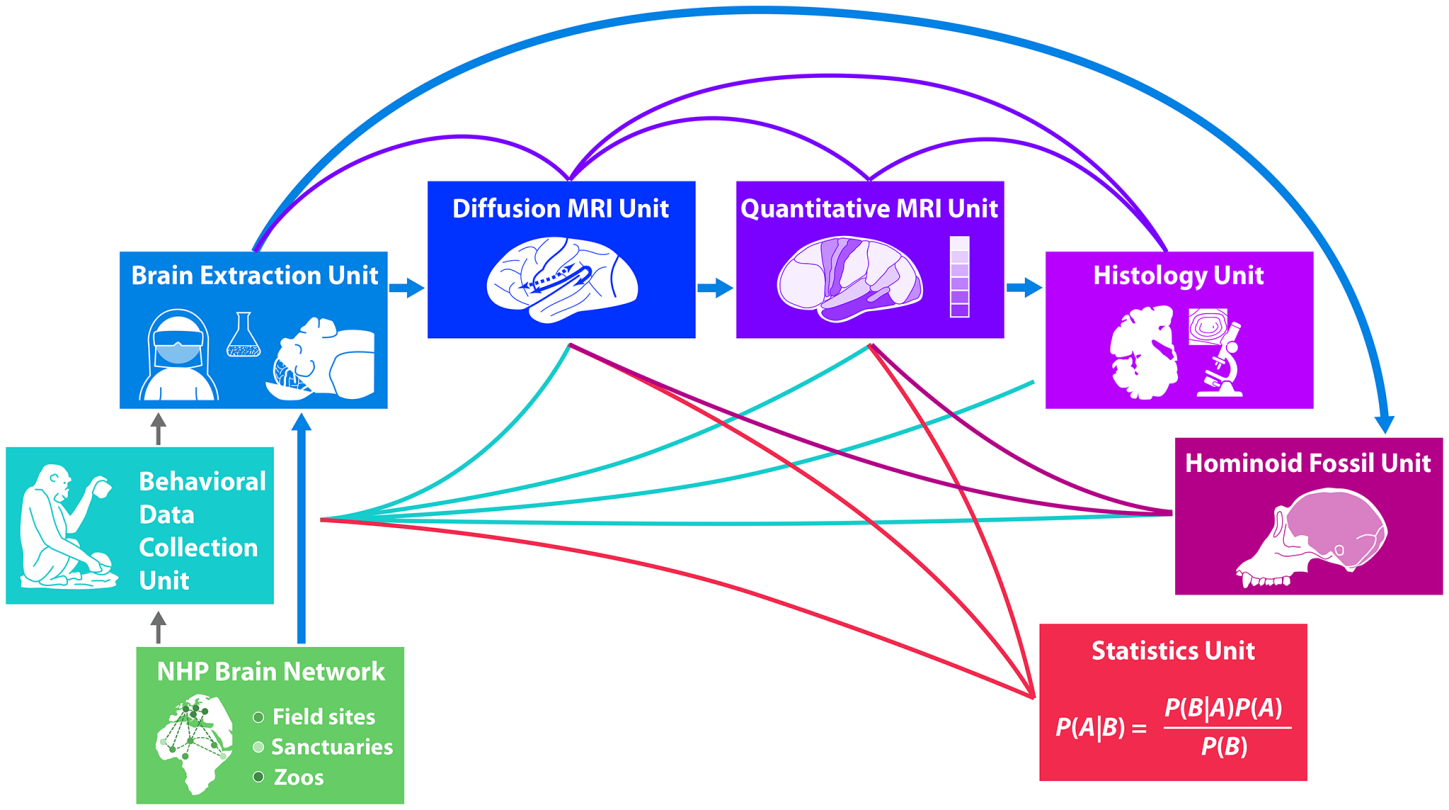
The boxes in this figure represent the different habitat sites of the animals (green flat boxes) and the different units of experts working in the project from *in vivo* behavior (turquoise box) before brain extraction (blue box) to *post mortem* macro-, micro-structural and histological analyses of the brain (dark blue to purple boxes) and to analysis of the endocast (dark purple box). The strong blue arrows indicate the transition of the brains after extraction to the respective brain analysis units in a sequential order and the animals’ skulls to the hominoid fossil unit. Green lines indicate possible correlations of *in vivo* behavioral data with *post mortem* brain analysis data. Purple lines indicate correlations between different brain structural analyses. Dark purple lines to the hominoid fossil unit (dark purple box) indicate correlations between gyral structure of the brain and the skull shape. The statistical unit (red box) provides methods to allow analyses of predictions under the condition of missing data points in the respective measurements which are based on input from the behavioral unit and from the brain analysis units, indicated by red lines.

In the following we first present the general methodological approach with its different research units schematically (Figure 1). We then describe the different methods used for each research unit. Given the vast differences between the different methods used, we cite the relevant literature for each method separately for each unit. Moreover, we present a validation of the method used in each research unit with a separate data set on individual chimpanzees from our non-human primate brain network.

The interdisciplinary Evolution of Brain Connectivity (EBC) consortium supporting the methodological approach discussed here encompasses researchers from seven different research units that work together using the pipeline displayed in Figure 1, spanning from *in vivo* behavioral data to *post mortem* brain and skull analyses. It starts with behavioral data collection of key populations, groups and individuals of NHP at different African field sites, sanctuaries and European Zoos in a large NHP Brain Network. Brain and skull extractions from naturally deceased NHPs are sourced from individuals of this large non-human primate brain network. Brains then enter the pipeline for brain structure analysis, starting with neuroimaging using advanced diffusion and quantitative MRI techniques, passing into histology with micro- and ultra-structural microscopical analysis. Skulls enter the pipeline in the hominoid fossil unit, where state-of-the-art scans reconstruct endocasts from the skulls using techniques developed with fossils. Finally, predictive Bayesian statistics integrate behavioral data with brain structure and endocast structure, allowing to deal with missing data. The uniqueness of the research using the present pipeline lies in the predictive power of *in vivo* behavior on *post mortem* brain structure. Weaving together different lines of evidence, the seven research units provide novel data to address long-standing questions concerning within-species and between species variation, and the evolution of hominoid brain structure and function. Thus far only first results from units early in the pipeline are available and published. These are behavioral data (Kopp et al., 2021; Girard-Buttoz et al., 2022a,b; Grampp et al., 2023; Bortolato et al., 2023a,b), data on brain extraction (Gräßle et al., 2023) as well as first detailed neuroanatomical data on a chimpanzee’s brain (Eichner et al., 2023). Results of later units in the pipeline inherently will only be available later.

## Methods and applications

### NHP brain network unit

**Figure 2:**
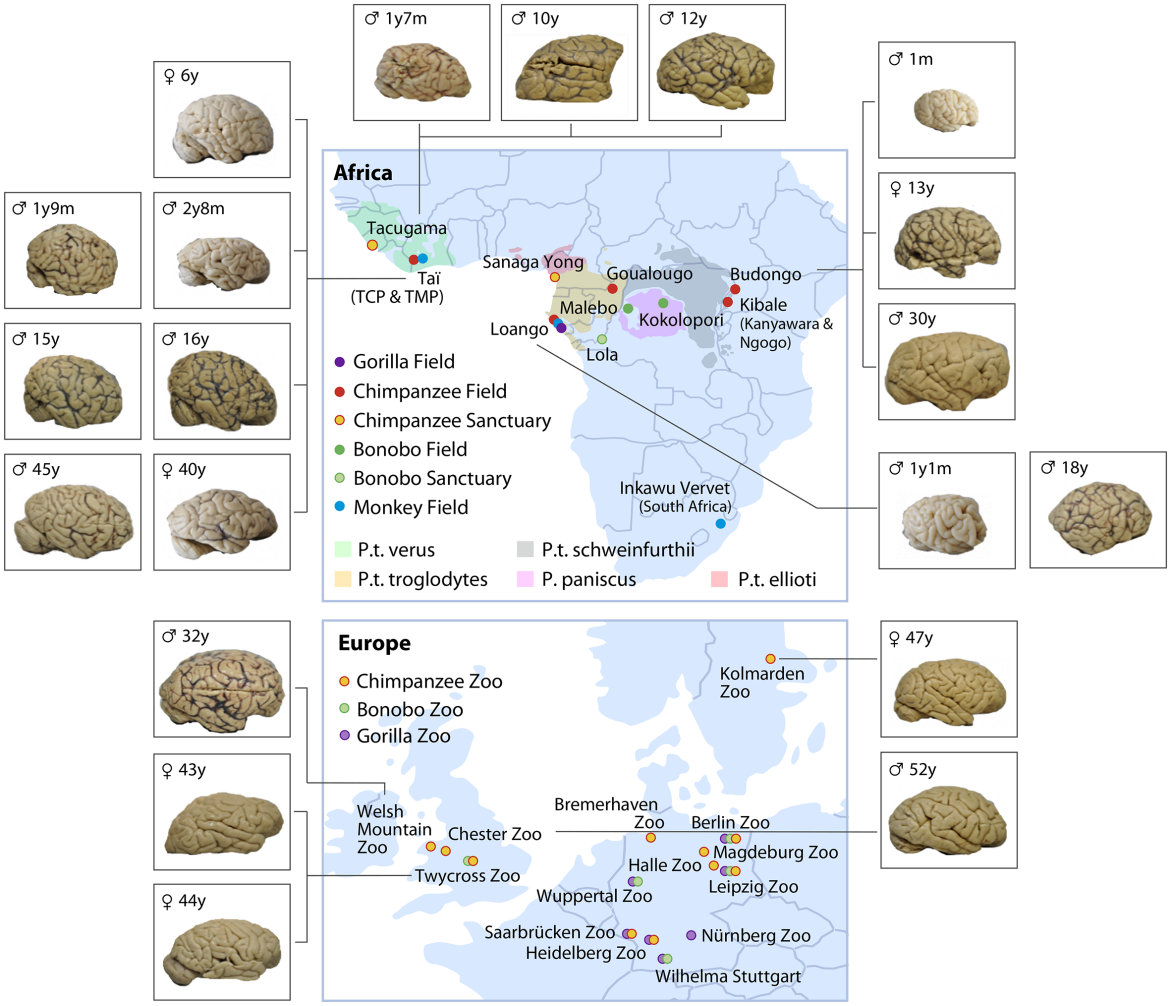
Non-human primate brain network consisting of ape and monkey field sites (unmarked Capuchins at Taboga, Costa Rica) and sanctuaries, as well as European zoos caring for great apes (color coded for species and ecology). The brains represent a subset of the collection of chimpanzee brains (sorted by age and site) which were collected between 2019 and 2022. The complete set of brains collected are listed in Table 1.

With the goal to sample key primate populations with different socio-ecologies, we established a large *NHP Brain Network* (Figure 2) involving great ape and monkey field sites, sanctuaries and zoos. To assess the scope of within-species variation, our network encompasses chimpanzee populations raised in highly different ecological and social conditions. For example, we have obtained brains from wild chimpanzee populations known to be exceptional tool users as well as from populations with limited tool use. Likewise, our network across zoos and sanctuaries includes chimpanzees with exposure to varying social and environmental factors, with the potential to influence communication and tool use. These between-population differences allow us to relate variation in environment and experience to variation in brain structure and behavior. To give scope for phylogenetic comparison, we extend this network to include other ape and monkey species from wild, zoo and sanctuary environments, some with known communication and tool use capacities (see Table 1).

**Table 1:** Number of brains per species acquired from various socio-ecologies (as of end 2022).

Species	Wild	Zoo	Sanctuary	Total
*Cebus capucinus*	2			2
*Cercocebus atys*	5			5
*Cercopithecus diana*	3			3
*Chlorocebus pygerythrus*	3			3
*Colobus polykomos*	2			2
*Gorilla gorilla*		4		4
*Pan paniscus*		4		4
*Pan troglodytes*	15	11	7	33
*Papio papio*		1		1
*Piliocolobus badius*	4			5
*Pongo abelii*		1		1
*Pongo pygmaeus*		1		1
Total	34	22	7	64

Partners from the NHP Brain Network, in collaboration with the experts from the EBC consortium, extract brains after unavoidable death by following procedures which ensure exceptionally high-quality MRI and histological data, with tissue quality validation demonstrated (Gräßle et al., 2023). Collecting *post mortem* brains from apes in a captive setting is already challenging,^1^ but collecting brains from wild primates with tissue quality suitable for high-end neuroimaging has rarely been achieved beforehand (Sherwood et al., 2004).

### Behavioral data collection unit

A core goal is to elucidate the possible function of brain networks, their specific neuroanatomical structure, their white matter pathways as well as their target regions. Hence, we assess the behavior of individuals for whom we may later examine the brains. This is a challenge since the EBC approach is non-invasive, and brains are extracted from individuals after natural or unavoidable death.

### Acquisition of individual demographic, life history, and ecological data

For each individual, we collect demographic and life history data that may impact the development and expression of cognitive abilities, behaviors, and associated brain structures. These variables include, e.g., subspecies, age, rearing history, reproductive history, group size and composition at different life stages. We use available sources, demographic and genetic records, video and audio recordings, publications and unpublished data provided by collaborators from field projects, zoos and sanctuaries. Ecological variables include habitat type and level of provisioning. Moreover, we developed a standard questionnaire for our collaboration partners at the zoos and sanctuaries to fill in for deceased individuals for whom we sourced the brain before behavioral data collection was possible. In addition to demographic and life history data, the questionnaire asks for retrospective assessments of specific behavioral characteristics, e.g., hand preference, tool use, and communication skills.

### Behavioral data collection at field sites

We use a “broad net” approach to acquire several types of behavioral data (e.g., vocalizations, tool use, social behavior), through the assessment of large numbers of individuals, thereby capturing variability across ontogeny (Estienne et al., 2019; Bründl et al., 2021), populations (Whiten et al., 1999; Gruber et al., 2009; Girard-Buttoz et al., 2022b) and species (see review in Girard-Buttoz et al., 2022a). To achieve this, first, data are collected from individuals using standardized protocols across some sites in the brain network. We target sites to maximize behavioral variation in the sample, specifically those with substantial differences in behaviors of interest, in socio-ecology or demography. Second, cross-site collaborations offer access to long-term demographic, genetic, behavioral and life history records, particularly valuable in long-lived species. Third, published studies provide relevant information on behavioral variation across a range of sites, such as in tool use (e.g., Whiten et al., 1999; Gruber et al., 2009) and social (e.g., Wilson et al., 2014; Surbeck et al., 2019) behavior.

**Figure 3:**
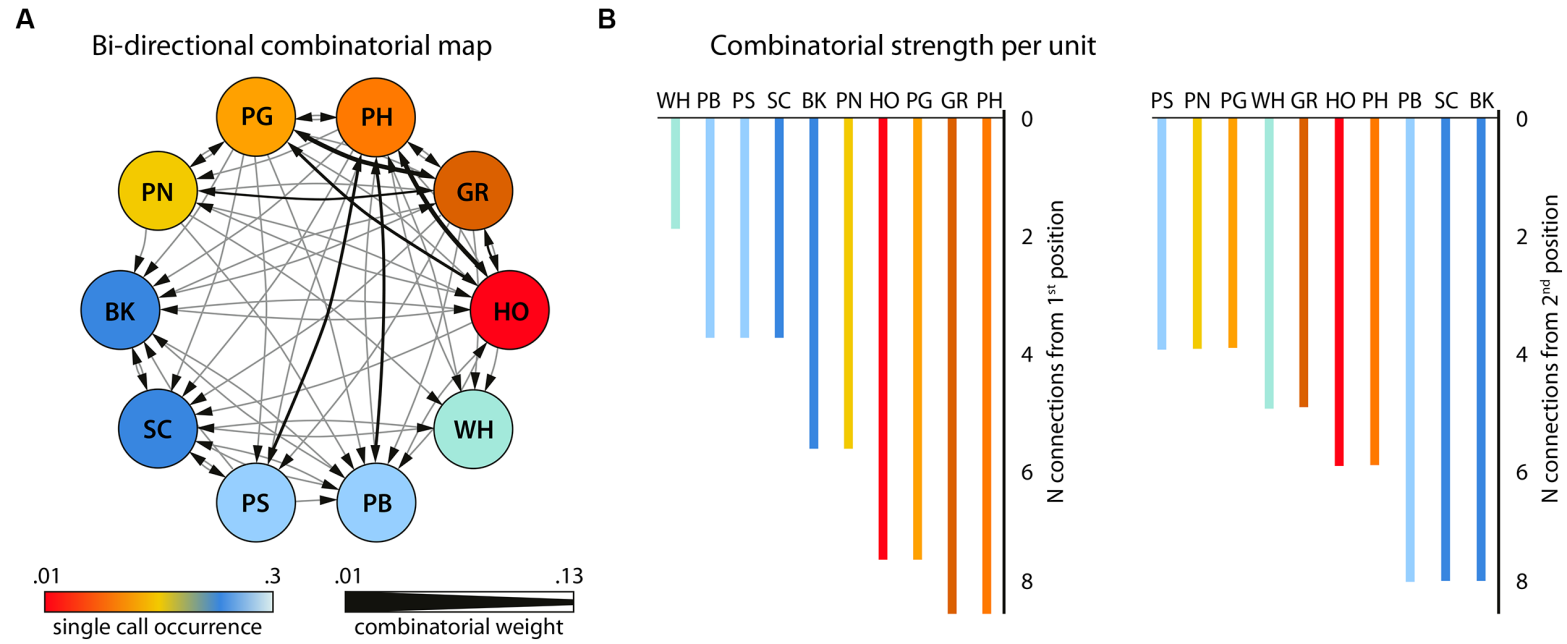
Bigram flexibility and ordering (*N* = 817 bigrams and 58 unique bigrams). **(A)** the bigram combinatorial network with the 10 single units that were combined with one other unit, depicted as circled nodes. Color gradients (hot-to-cold) represent the number of times a certain unit is found in the bigram set. Note that the single units (NV) and (PR) that never occurred in bigrams in this dataset are not shown here. This network was also used for the calculation of the Betweenness Centrality among the units. The size of the directional edges (arrows) expresses the number of times the specific bigram is found in the sample (thick-to-thin). **(B)** The number of different single units with which each single unit forms a bigram in the sample as first unit (left) or second unit in the bigram (right). Adapted from Girard-Buttoz et al. (2022a).

Focusing on vocal behavior as an example, to date we analyzed large datasets of recordings of utterances of wild adult chimpanzees from Tai National Park with respect to positional regularities in vocal sequences (Girard-Buttoz et al., 2022a). We found systematic regularities in two-unit and three-unit sequences (bigrams and trigrams, respectively), and their relative position, first position or final position (Figure 3). This systematicity may be viewed as a first step toward structured sequences, known to be crucial for language, and is amenable to examining ontogenetic development, individual, population and species differences. For example, vocal sequence length and diversity expands slowly but considerably across development, reaching adult levels at 8–10 years old (Bortolato et al., 2023a). Also, systematic population differences in call order, associated with different socio-ecologies, are consistent with call sequence usage learning (Girard-Buttoz et al., 2022b). A broad review across primate species highlights that a variable number of vocal sequences are described per species, and are considerably fewer than those found in chimpanzees (Girard-Buttoz et al., 2022a). Quantitative analyses across species using the same data collection protocols are required to establish direct comparisons between species. For example, a within- and between-species study, using the same data collection protocols across species, supports the idea that socio-ecological drivers such as fission-fusion structures and social uncertainty impact communication complexity (Grampp et al., 2023). Such comparative quantitative data can aid formulation of hypotheses for relevant brain pathways.

In general, our large-scale data collection strategy offers the best likelihood that brain samples are from known individuals or populations with known behavior across all ages, offering assessment of behavioral capacity across four axes: species, populations, individuals and ontogeny.

All field sites, which collaborate in the NHP brain network, follow site specific protocols for data collection. One example for a field site protocol is the long-term data collection protocol employed by the Tai Chimpanzee Project (Wittig and Boesch, 2019). Here trained research assistants observe the behavior of chimpanzees during full day follows using focal animal or scan sampling. Protocols systematically document aggressive and affiliative social parameters, tool-use and vocalizations.

In addition, certain research projects use specifically designed protocols in relation to topics of interest for the pipeline, e.g., vocal communication (Girard-Buttoz et al., 2022a,b) or tool use (similar to Luncz et al., 2012; Sirianni et al., 2015). Across some field sites, the same protocol is either routinely used (social behavior, Surbeck et al., 2021) or conducted for certain projects (vocal and social behavior, Fedurek et al., 2021). In addition to observational data, field experiments address specific questions in the cognitive domain (Zuberbühler and Wittig, 2011; Wittig and Crockford, 2018). Previous experiments, for example, have been conducted across populations (tool use, Gruber et al., 2009) and species (Mielke et al., 2019; Girard-Buttoz et al., 2020).

### Behavioral data collection at zoos and sanctuaries

We developed a protocol applicable in various settings to obtain comparable data across highly varied housing facilities in zoos and sanctuaries. It combines standard observation methods, audio and video recordings, and easy-to-perform behavioral and cognitive tests. To ensure data quality, all observers and coders receive intensive training and must pass a test for inter-rater reliability with good agreement (Cohen, 1960).

We assess individuals’ behavioral and communication repertoire and social integration based on data collected through randomized 30 min focal observations (Altmann, 1974) over several days (minimum: 10 h per individual in total), covering daily activity time. Behavioral data are continuously recorded and live coded using the CyberTracker application on smartphones.^2^ The implemented ethogram comprises the individual’s activity, social interactions, including, e. g., grooming, social play, aggression, post-conflict behavior, social support, infant care, vocalizations, gestures, tool use, and proximity to others. It is adapted from ethograms used within the Taï Chimpanzee Project, allowing for cross-site comparison. Simultaneously, we record vocalizations using a digital recorder with a directional microphone and complex social interactions using a digital video camera during focal observations. These recordings enable detailed and reproducible analyses afterwards. Spectrograms of recorded vocalizations are subsequently analyzed using PRAAT (Boersma and Weenink, 2022). Proximity data are obtained by instantaneous sampling each 10 min during a focal observation (Altmann, 1974). Here, we indicate the nearest neighbor and the individuals in body contact, within arm’s reach and 5 m range relative to the focal individual. In addition to focal observations, we aim to record vocalizations whenever feasible, and the caller identity is determinable (*ad libitum*) (Altmann, 1974).

For a detailed study of particular abilities and behaviors, we conduct cognitive and behavioral tests applicable in the usual social setting (e.g., on mirror self-recognition using shatterproof hand mirrors, Kopp et al., 2021). All tests are continuously video-recorded from several perspectives and subsequently video-coded using coding schemes implemented in BORIS (Friard and Gamba, 2016). Vocalizations are additionally analyzed using PRAAT (Boersma and Weenink, 2022). Depending on the respective research question, the data are analyzed on an individual, dyadic, population, or species level and from an ontogenetic perspective.

Focusing on tool use skills as an example, to assess an individual’s tool use and manufacture skills, functional knowledge, and hand preference, we attach transparent dipping tubes filled with diluted juice to structures in the enclosure and provide sets of differently efficient tools (N_ToolDippingSet_ = N_Individuals_). Several observers film all interactions related to the task until the tubes are empty. Based on these videos, we code, e.g., all actions related to handling the tubes and tools, tool type, hand used, and tool use success, using a detailed coding scheme (Ebel et al., personal communication). Dipping tasks are frequently used as behavioral enrichment in zoos and sanctuaries, triggering tool use behaviors common in natural populations, allowing sound comparisons across sites.

### Brain extraction unit

*In-toto* extracted, structural intact brains exhibiting preserved cellular architecture pose the prerequisite for obtaining meaningful data via state-of-the-art imaging techniques as applied within the project’s framework. We successfully implemented an extraction and fixation protocol across collaborative sites resulting in high-tissue quality specimens from deceased apes regardless of their origin—including zoo-housed and wild roaming individuals (for a detailed description please view, Gräßle et al., 2023). In brief, brain removal is attempted as soon as possible following the individual’s death as *post mortem* lytic processes progressively deteriorate tissue integrity. This is particularly of concern under hot conditions found in the ape’s natural habitat of tropical African rainforests (Tsokos and Byard, 2012). There, an early response to an ape fatality is achieved by daily follows of habituated ape groups combined with a close health monitoring that is coordinated by an on-site veterinarian. The field veterinarians are further responsible for conducting necropsies and brain removal at the location of the animal’s death, possibly deep inside the rainforest. Necropsies are performed under strict hygiene protocols including full personal protective equipment as the ape might have succumbed to a severe zoonotic disease (Patrono et al., 2020).

The brain removal is conducted with the primate positioned in dorsal recumbency and a forward flexed head. After soft tissue has been dissected, the cranium is opened utilizing an oscillating saw. Landmarks for the cut are the upsloping frontal bone behind the supraorbital ridge and the occipital bone just below the occipital protuberance. Anchoring basal brain structures (cranial nerves, vascular supply) are visualized and severed from rostral to caudal, while maintaining a steady, slight backwards-pull on the skullcap. After cutting the medulla oblongata the skullcap containing the brain is fully detached and a string is threaded underneath the basilar artery (see below). Subsequently the brain is bluntly mobilized from the skullcap and directly transferred into 10% neutral buffered formalin (NBF), neutrally buffered to avoid brain deformations due to osmotic pressure changes. To reduce the chance of plastic deformation during fixation of the extracted brains, they are submerged in formalin with the top side facing downward, while they remain tethered to the string. Fixation is conducted at a cool temperature (~4°C) for the first t2 weeks and the formalin is exchanged after 24 h and after a total of 7 days. Fixation is performed on floating brains to minimize brain deformation during fixation process. At the 14th day the formalin is renewed again and the brain is stored at ambient temperature with regular changes of the fixative until the export can be conducted. Heads are either directly immersed in 10% formalin (applicable for small/young individuals) for disinfection, or defleshed via burying them in the ground for several months. Once fixed, brain specimens are shipped to the Max Planck Institute for Human Cognitive and Brain Sciences where they are bio-banked, and skulls are exported to the Max Planck Institute for Evolutionary Anthropology, both situated in Leipzig, Germany.

The first ever extracted complete wild chimpanzee brain from a six-year-old chimpanzee in the Taï National Park, Côte d’Ivoire was collected 4 h *post mortem*, due to the dedication of observers and field veterinarians (Gräßle et al., 2023) and analyzed using MRI (Eichner et al., 2020).

### Diffusion MRI unit

#### Diffusion MRI data acquisition

The close interaction between different cortical areas via white matter fiber pathways in the brain is a central neurobiological basis of complex cognitive tasks in primates. Diffusion MRI (dMRI) tractography is an established method to reconstruct structural connections in specialized networks (Perani et al., 2011). In our prior work we showed that the maturation of the dorsal fiber tract targeting the posterior portion of Broca’s area (Brodmann area 44) in the inferior frontal gyrus as part of the language network correlated with the ability to process complex sentences (Skeide et al., 2016). Comparison of this network with homolog connections in apes and monkeys allows an investigation of the differences in this neural network and its homolog structures across primates.

Here, whole-brain diffusion MRI data (dMRI) were acquired on a preclinical Bruker Biospec 94/30 MRI system at 9.4 T (Software Version Paravision 6.0.1), using a 300 mT/m gradient system and a 154 mm transmit-receive quadrature RF coil (Bruker BioSpin, Ettlingen, Germany). Diffusion MRI Data were acquired using a spin-echo segmented 3D EPI sequence. To minimize the impact of B1+ inhomogeneity across the whole brain volume, the sequence utilized two adiabatic refocusing pulses. Double EPI sampling was utilized to mitigate Nyquist ghosting artifacts (Yang et al., 1996). Data with an isotropic resolution of 500 μm were acquired using the following parameters: TR = 1,000 ms, TE = 58.9 ms, matrix: 240 × 192 × 144, no partial Fourier, no parallel acceleration, EPI segmentation factor = 32, EPI BW = 400 kHz. Diffusion MRI data were acquired using a diffusion weighting of b = 5,000 s/mm^2^ in 55 directions, spread across a full sphere. Prior to the acquisition, 10 diffusion-weighted volumes were acquired as dummy scans to achieve a constant steady-state sample temperature. Three interspersed *b* = 0 images without diffusion weighting were acquired for drift correction. To correct for off-resonance EPI distortions, an additional *b* = 0 volume was included with the phase encoding direction reversed. A noise map with matching EPI parameters was additionally recorded to characterize the noise statistics of the dMRI data. The total dMRI acquisition time was approximately 90 h.

#### Diffusion MRI data processing

Diffusion MRI preprocessing included the following steps: (i) Signal debiasing utilizing the acquired noise map (Gudbjartsson and Patz, 1995; St-Jean et al., 2020). (ii) MP PCA Denoising (Cordero-Grande et al., 2019). (iii) 3D Gibbs Correction using sub voxel shift (Kellner et al., 2016). (iv) Linear signal drift correction (Vos et al., 2017). (v) Correction of eddy currents and EPI distortions (Andersson and Sotiropoulos, 2016). Initial deterministic whole-brain diffusion tensor imaging (DTI) tractography was performed using the software brainGL.

**Figure 4:**
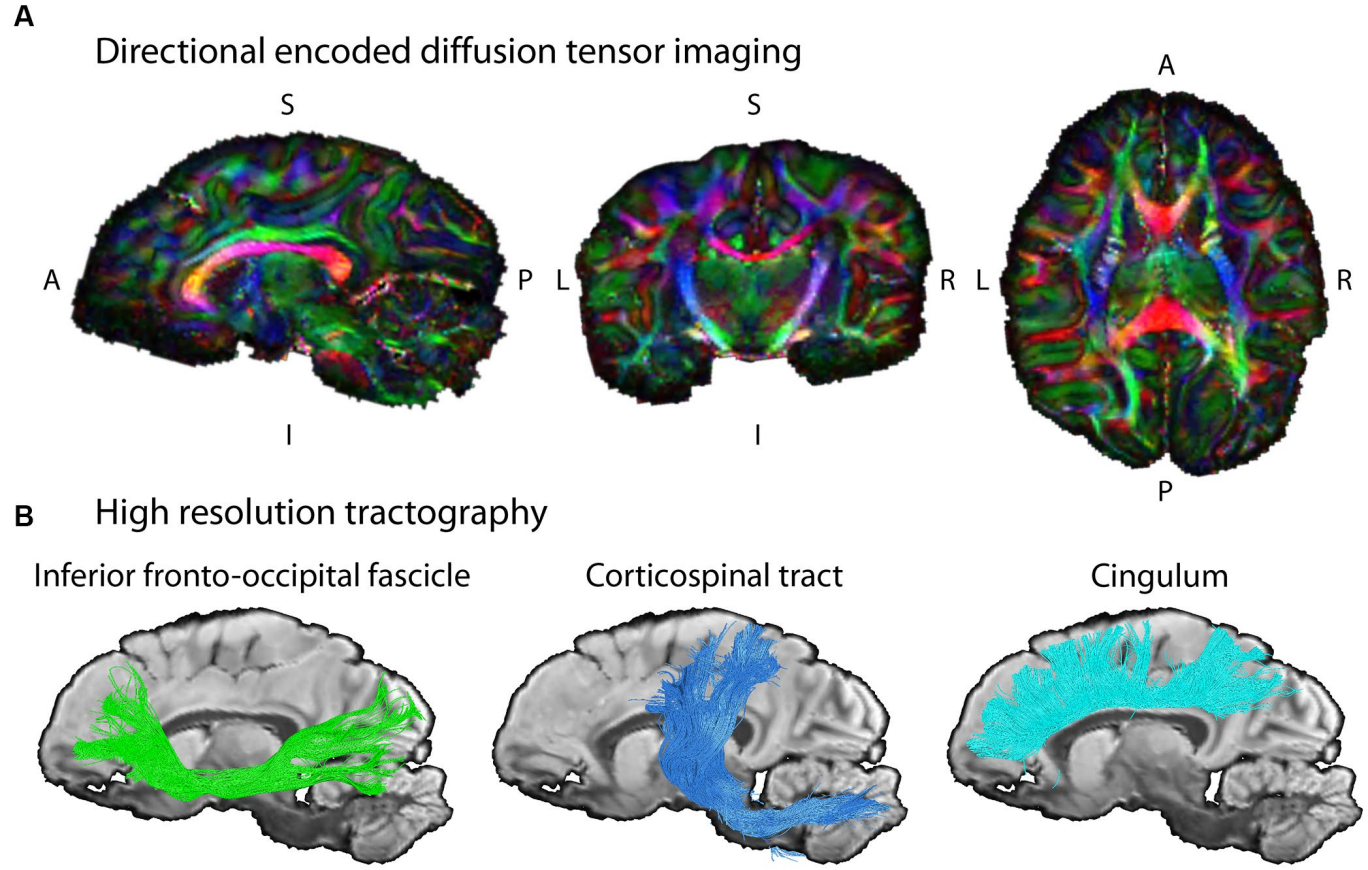
Example diffusion MRI reconstruction and tracking results. **(A)** The unprecedented data quality with ultra-high resolution allows for mapping the structural connectivity of the chimpanzee brain (wild, female, 6 years) with high precision. The images shows slices of the color coded fractional anisotropy computed from the measurement (color coding of main diffusion direction: red—left right, green—superior inferior, blue – anterior posterior). **(B)** The high-resolution dMRI data enables tractography on fine spatial scales in the same chimpanzee brain. Three respective tract reconstructions are depicted: Inferior Fronto-Occipital Fasciculus, IFOF (green); Corticospinal tract (blue); Cingulum (turquoise). The three depicted tracts were selected as examples for this illustration due to their well-known morphology in humans.

Within the present project, we have developed a method for *post mortem* dMRI of brains of wild living and captive animals with ultra-high spatial resolution and unprecedented quality leveraging specialized imaging sequences, a dedicated high-performance MRI system, and extended measurements over multiple days (Eichner et al., 2020, accepted for publication). by very long measurements in a dedicated high-performance MRI system (Eichner et al., 2020, accepted for publication). The data acquired in this project allow for analyzing the development and ontogeny of the white matter fiber pathways in the brain with highest precision in the individual animal and demonstrated here for a single chimpanzee from our NHP brain network (Figure 4). This further allows to compare the network properties between groups and species in relation to the behavioral differences.

#### Quantitative MRI unit

Quantitative MRI allows us to obtain physical parameters of brain tissue, which are sensitive biomarkers of brain microstructural properties including myelination and iron content (Weiskopf et al., 2021). The method is a powerful approach to study whole-brain cortical myelination patterns, brain organization, development, and brain iron accumulation with age. When used at ultra-high spatial resolutions at ultra-high fields it captures finest details of brain anatomy including cortical myeloarchitecture and highly myelinated short association fibers (Kirilina et al., 2020; Weiskopf et al., 2021).

#### QMRI acquisition

Brains were immersed in the proton-free solution Fomblin (Solvay) in an egg-shaped acrylic container and scanned on a human whole-body 7 T Terra MRI scanner (Siemens Healthineers, Erlangen, Germany), using a 32-channel receive 1-channel transmit radio-frequency (RF) human head coil (Nova Medical, Wilmington, MA). Multi-parametric mapping was implemented using a multi-echo 3D FLASH sequence (Weiskopf et al., 2013; Vaculčiaková et al., 2022) at 300 μm isotropic resolution [matrix: 432 × 378 × 288; readout bandwidth of 331 Hz/pixel; repetition time TR = 70 ms; 12 equidistant echoes between echo times (TE) 3.63 ms and 41.7 ms with a spacing of △TE = 3.56 ms; excitation flip angles: 18° (PD and MT-weighted), 84° (T1-weighted); MT pulse: Gaussian at 3 kHz offset, flip angle: 700°]. The receive sensitivity (B1^−^) field was partly corrected using the ratio of low resolution (2.1 mm) T1-weighted images acquired with the receive coil over images acquired with the transmit coil. A simultaneous spin and stimulated echo 3D-EPI sequence was used to obtain maps of the RF transmit field B1^+^ and a double echo gradient echo sequence was used to obtain a map of the static magnetic field B_0_ (which was used to correct the B1^+^ map for EPI distortions) (Lutti et al., 2010, 2012). B1^+^ mapping was done at an isotropic resolution of 4 mm, with a matrix size of 48 × 64 × 48 and the following acquisition parameters: TR = 500 ms; TE = 40.54 ms; GRAPPA acceleration factor = 2 × 2. B_0_ mapping was done at an isotropic resolution of 2 mm and a matrix size of 96 × 96 × 64, with the following acquisition parameters: TR = 1,020 ms, TE = 10 and 11.02 ms, flip angle = 20°. The temperature of the sample was stabilized around 30° by warm air flow directed onto the sample and was monitored by a temperature sensor (Luxtron, Advanced Energy).

#### QMRI analysis

The quantitative maps were computed with the open source hMRI toolbox^3^ (Tabelow et al., 2019). B1^+^ and B1^−^ maps were processed with boundary-preserving smoothing with a Gaussian kernel of 8 mm. After distortion correction of the individual echoes of the weighted images (Weiskopf et al., 2005; Ruthotto et al., 2012), the effective transverse relaxation rate (R2*) was fit using weighted least squares. From this fit, the weighted images were extrapolated to TE = 0 ms. These images were then used to calculate MT_sat_ as described in Helms et al. (2008) and R1 as described in Dathe and Helms (2010) and proton density PD was calculated as the signal amplitude described by Dathe and Helms (2010). The MT_sat_ images were additionally B1^+^ bias corrected (Lipp et al., 2023).

**Figure 5:**
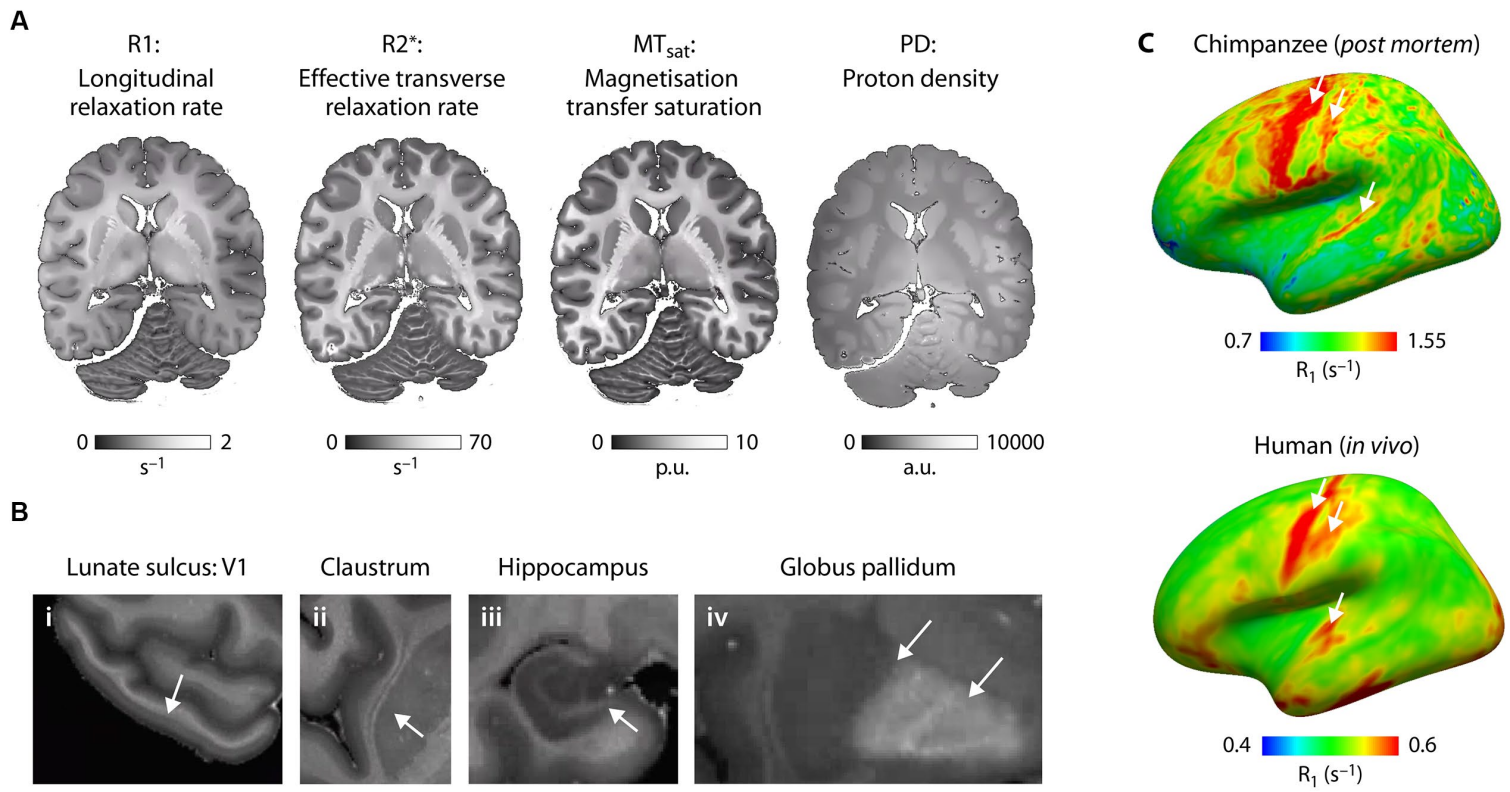
Microstructural mapping of a chimpanzee brain using quantitative MRI. **(A)** The quantitative MRI parameters R1, R2*, MT_sat_ and PD acquired at 7 T and ultra-high resolution are shown for the same axial slice of one *post mortem* chimpanzee brain (wild, male, 45 years.). All parameters show exquisite contrast between gray and white matter and are sensitive biomarkers of brain tissue microstructure including brain myelination and brain iron content. **(B)** The high resolution multimodal maps allow for comprehensive characterization of brain anatomy resolving smallest neuroanatomical features including (i) layers within the cortex (arrow indicates the highly myelinated Stria of Gennari in the primary visual cortex in the lunate sulcus), (ii) thin gray matter structures such as the claustrum, (iii) substructures within the hippocampus and (iv) iron accumulation in subcortical structures. **(C)** Whole brain quantitative MRI maps allow for mapping of myeloarchitecture across the entire brain facilitating quantitative comparison of brain organization across species. R1 sampled at middle cortical depth and mapped across the neocortex for (bottom panel) one *post mortem* chimpanzee brain (captive, female, 44 years.) reflect different myelination of brain areas (highly myelinated motor, sensory and auditory cortex indicated with arrows) and can be compared with the same metric obtained in (right panel) humans *in vivo* (Kirilina et al., 2020). Note that absolute R1 values are larger due to fixation of the *post mortem* brain tissue compared to *in vivo*.

As shown here for an individual chimpanzee from our NHP brain network, we have established cutting-edge multiparametric quantitative MRI acquisition and analysis methods to study *post mortem* primate brains at a field strength of 7 T and at unprecedented ultra-high resolution of 300 μm, enabling imaging of submillimeter structures including cortical layers and subcortical nuclei (Figure 5). We obtain quantitative maps of four crucial MR parameters: proton density (PD), longitudinal (R1) and effective transverse (R2*) relaxation rates, and magnetization transfer saturation (MT_sat_). All maps (Figure 5) provide excellent gray-white matter contrast and reflect brain myelination (PD, R1 and MT_sat_), cortical myeloarchitecture (all) and brain iron content (R2*) (Weiskopf et al., 2021). They enable high quality segmentation of cortical and subcortical structures on individual level and co-registration to existing brain atlases.

This project is the first to obtain quantitative whole-brain myelination biomarkers in great apes. Previous studies on the chimpanzee brain have been limited to a single semi-quantitative indicator of cortical myelination (excluding white matter), the T1w/T2w ratio. It was obtained from anesthetized captive chimpanzees at comparatively low field strength (3 T) and low resolution (19 times larger voxel volume as compared to our data) (van Essen et al., 2018). Our dataset allows to capture multiple key characteristics of the primate cortex including cortical thickness and cortical myeloarchitecture across brain areas, across development and across species.

### Histology unit

**Figure 6:**
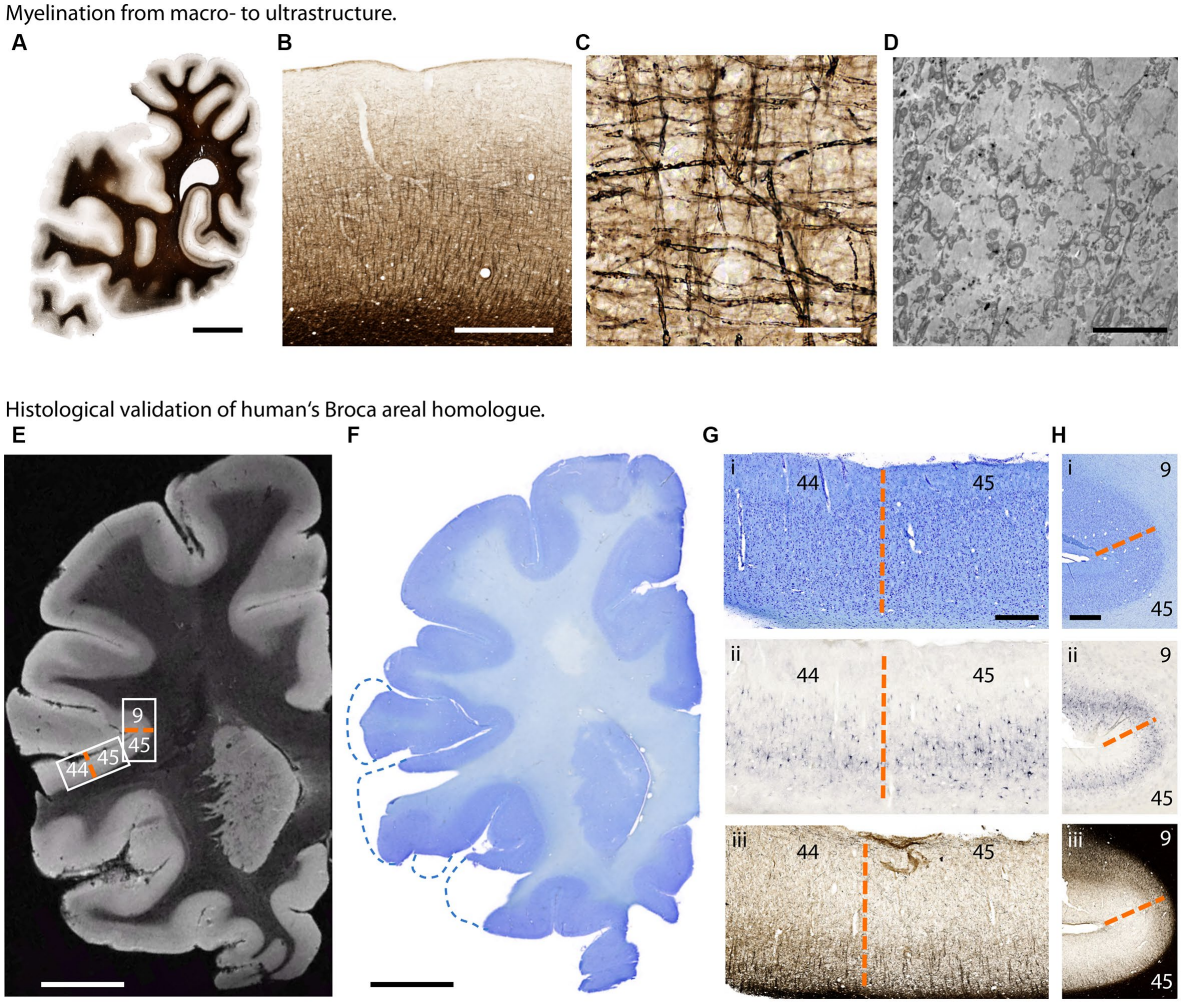
Histological investigation of great ape brain samples. **(A–D)** Myelination from macro- to ultrastructure in a chimpanzee brain. **(A)** Section of one hemisphere illustrating myelin distribution (Gallyas silver stain). **(B)** Cortical myelination profile (Gallyas). (C) Cortical myelinated fibers in detail (Gallyas). **(D)** Myelinated axons in electron microscopy. Scalebars: **(A)** 10 mm, **(B)** 1 mm, **(C)** 50 μm, **(D)** 5 μm. **(E–H)** Histological validation of human’s Broca areal homolog in a bonobo brain. **(E)** T2*-weighted MR image acquired at 50 μm isotropic resolution. Low intracortical myelination in areas BA 44 and 45 results in higher image intensity. **(F)** Corresponding Nissl stained section. **(G)** Border between Brodmann area 44 and 45 homologs (dashed orange line) in (i) Nissl stain for cytoarchitecture, (ii) Aggrecan stain for neuronal extracellular matrix and (iii) Gallyas silver stain for myelin. **(H)** Border between Brodmann areas 45 and 9 homologs (dashed orange line) in (i) Nissl, (ii) Aggrecan and (iii) Gallyas. Scalebars: **(E,F)** 10 mm, **(G)** 1 mm, **(H)** 500 μm.

Expansion of brain size and complexity within the hominoid lineage goes hand in hand with the changes in brain cellular organization. The cytoarchitectonic difference between the posterior portion of Broca’s area [Brodmann area (BA) 44] and its anterior portion (BA 45) has been discussed as relevant parts of the human language network. At the macroscopic level, qMRI and dMRI unravel cortical myelo-architecture and white matter connectivity across brain regions. However, due to the constraints in spatial resolution these techniques provide only limited insights into the brain structure at the cellular, subcellular and ultrastructural level (van Essen et al., 2018). Advanced neurohistology is the key method to deliver comprehensive microstructural information both in 2D and 3D (Morawski et al., 2018) by the combination of gold standard histochemistry and immunohistochemistry using a broad range of available stains and antibodies (Figures 6A–D; Kirilina et al., 2020).

For state-of-the-art histology whole brains were cut in 15 mm thick coronal slabs and cryoprotected in 30% sucrose in PBS pH 7.4 containing 0.1% sodium azide. Entire hemispheres were cut on a cryomicrotome into 30 μm sections (Thermo Scientific, Microm HM430, freezing unit Thermo Scientific, Microm KS34). Sections were collected in sealed sample glasses in PBS pH 7.4 containing 0.1% sodium azide and stored until further use at 4°C. Consecutive sections were stained with histological and immunohistochemical methods. To facilitate co-registration between MRI and histology, block-face imaging was applied during sectioning (Brammerloh et al., 2021).

To investigate the cortical cytoarchitecture, Nissl staining with acetate-buffered cresyl violet was applied. Visualization of myelin was performed using modified Gallyas stain or anti-myelin basic protein antibody (rat anti-MBP, 1:400, VWR, Germany), visualization of neuronal extracellular matrix was performed with anti-aggrecan antibody (HAG 7D4, 1:800, Origene, Germany).

Digital imaging of the entire object slides was performed at an AxioScan.Z1 (Zeiss, Jena, Germany) fully automated microscope with either a 20× 0.7 NA or a 40× 0.95NA Plan-Apochromate objective with ~400 nm, respectively, ~220 nm axial resolution.

For ultrastructural investigations small blocks of brain tissue were additionally fixed using fixative containing 3% formaldehyde and 1% glutaraldehyde in pH 7.4 buffered 0.2 M Cacodylate buffer. Vibratom sections (50 μm) were taken. Small tissue blocks were dissected from vibratome sections, post-fixed in buffered 1% osmium tetroxide at room temperature for 1 h, rinsed in cacodylate buffer, dehydrated in a graded series of acetone including a 1% uranyl acetate stain at 70% acetone for 30 min and embedded in Durcupan araldite casting resin (Carl Roth, Karlsruhe, Germany). For structural orientation, semithin sections were cut at 1 μm thickness and stained with toluidine blue. Ultrathin sections (~50 nm) were cut on an Ultracut II (Leica Microsystems, Wetzlar, Germany). Samples were imaged with a LEO-Zeiss EM 912 Omega TEM (Zeiss, Oberkochen, Germany) at 80 kV, and digital micrographs were obtained with a dual-speed 2 K-on-axis CCD camera-based YAG scintillator (TRS-Tröndle, Moorenweis, Germany). Data were obtained from different sections taken from different areas in 2 brains. Measurements were performed using ImageSP analysis Software (TRS-Tröndle, Moorenweis, Germany).

As demonstrated here, we established a histological pipeline combining block face imaging and 2D serial sections to achieve 3D mapping of cyto- and myelo-architecture with microscopic resolution, complementing microstructure mapping by dMRI and qMRI. Co-registration of qMRI (Brammerloh et al., 2021) and dMRI with 3D histology is achieved by a multi-step co-registration procedure, which includes the co-registration to histological 3D block face images as an intermediate step. The 3D histology is used to validate qMRI metrics of myelination and iron content and to characterize the cortical targets of white matter tracts identified by dMRI. For example, the anatomical location and borders of Broca’s area homologs are identified by Nissl stain revealing the cytoarchitecture, Gallyas silver stain capturing the myelo-architecture and immunohistochemistry with anti-aggrecan antibody visualizing the neuronal extracellular matrix and demarcating the boundaries between BA 44 and 45 homologs (Figures 6E–H). Thus, the present histological method provides a powerful tool to identify functionally-specialized areas in *post mortem* brain in the absence of functional neuroimaging data and allows to study for example the homologs of language-related white matter tracts in hominoid brains.

The described and established pipeline of advanced histological methods, applied on this exceptional collection of brains, allows (i) the qualified validation of qMRI and dMRI data with high resolution, (ii) detailed investigation of developmental aspects in comparison to humans, (iii) provides the microstructural basis of behavior by validating evolution, development, behavior and MR-neuroimaging with advanced histology we are bridging the gap to ground truth microanatomical connectivity.

### Hominoid fossil unit

**Figure 7:**
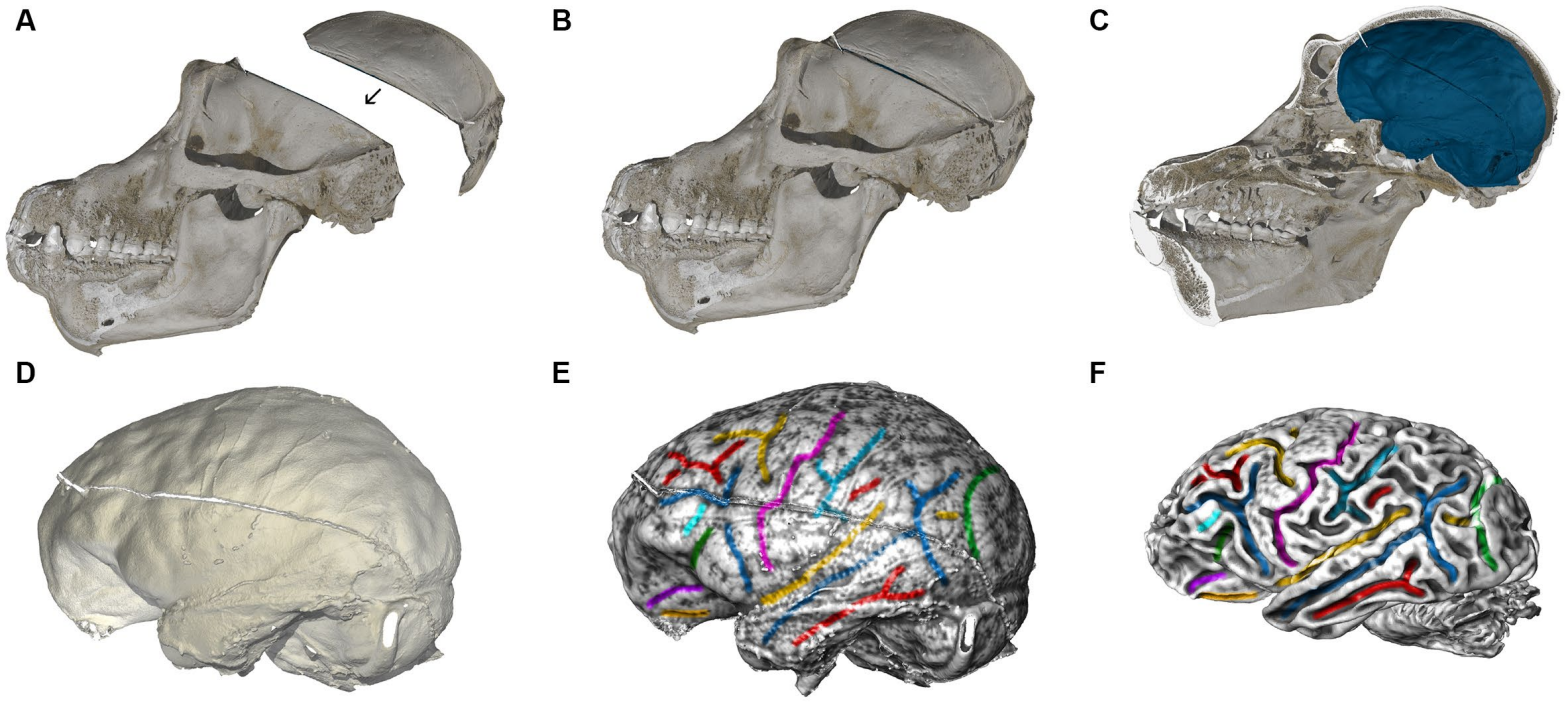
Computed tomographic (CT) and MRI scans of a chimpanzee. **(A)** Skull fragments are scanned using microCT and realigned virtually based on anatomical criteria **(B)**. **(C)** Segmentation of the interior braincase yields an endocranial imprint (blue). The skull is virtually cut in the midsagittal plane. **(D)** Impressions created by blood vessels, cranial sutures, gyri and sulci are clearly visible on the virtual endocast. **(E)** We visualized the shape index of the surface as a gray-level gradient to facilitate the identification of sulcal impressions, and then manually color-coded brain features corresponding between endocast and brain. **(F)** MRI brain scan of the same chimpanzee individual, analyzed by the Diffusion MRI Unit. Corresponding features on the endocranial imprint in **(E)** are shown in the same color.

The hominoid fossil unit aids in understanding the evolution of brain size and structure through endocranial imprints from the fossil record. We complement neuroimaging with micro-CT scans of skulls to examine the brain’s imprint (or endocast) on the cranial cavity. Comparative analyses of these endocasts from extant and extinct primates highlight evolutionary trends in brain volume and organization, including developmental rates (Gunz et al., 2020). Key organizational changes in hominins, such as in the prefrontal cortex (Holloway et al., 2018; Ponce de León et al., 2021), the parietal and cerebellum are of particular interest (Bruner, 2004; Neubauer et al., 2018; Pereira-Pedro et al., 2020). However, endocasts only capture the tissues surrounding the brain’s outer surface, and therefore only some aspects of brain organization. By combining MRI data with endocranial imprints of the same individuals, we can better understand neuroanatomical variations within and across species. Skulls were scanned using a Diondo d3 high-resolution micro-CT system at the Max Planck Institute for Evolutionary Anthropology in Leipzig. Small specimens (i.e., infants or monkeys) were scanned with a resolution of 15 μm (isotropic voxel size), large adult apes with a resolution of 90 μm. Micro-CT scans were performed either on bone after cleaning, specimens fixed in formaldehyde (or sometimes frozen heads when brains are extracted in zoos). As brain extraction requires opening the skull, all cranial fragments are micro-CT scanned separately, and then reassembled on the computer based on anatomical criteria (Figure 7) in the software VGStudio MAX (Version 3.5),^4^ following the cranial reconstruction protocols by Gunz et al. (2009, 2020). The same software was used to create virtual endocranial imprints following Neubauer et al. (2009): first the cranial bone was segmented automatically by defining a grayscale range. Next, the labelfield of the bone was dilated in 3D until small cranial foramina and sutures are closed. The foramen magnum was sealed manually. The endocranial cavity was then segmented automatically using a region growing algorithm. Then this endocranial segmentation was dilated by the same number of voxel layers used to expand the bone in the previous step. To facilitate the identification of gyri and sulci on the extracted endocranial surface we visualized its local surface topology as a grayscale gradient of the shape index in the software package Avizo (Version 2021; Thermo Fisher Scientific). Following Koenderink and van Doorn (1992) the shape index is based on the principal curvatures C1 and C2: 2/Pi*arctan*(C1 + C2)/(C1-C2).

As visualized in Figures 7E,F, almost all brain sulci leave unambiguous impressions in the endocranial cavity. Comparing endocranial shape (Figures 7C–E) with the brain scanned after extraction (Figure 7F) reveals subtle changes in overall shape, including a slight but noticeable reduction in its superior–inferior dimension. This phenomenon is likely related to the removal of the surrounding connective tissues, and the absence of cerebrospinal fluid in the ventricles. Fixation processes may further alter the brain’s shape. These effects should be considered when directly comparing brains scanned after extraction to brains scanned within the skull (both *in vivo*, and *post mortem*). Since the endocast reflects the pre-extraction shape of the brain along with its surrounding structures, we can use the endocranial surface as a template to correct for alterations to the brain’s shape that occur after extraction. Such corrections can be made, e.g., through algorithms designed for surface or semilandmark-based retrodeformation (Gunz et al., 2009).

### Statistics unit

One of our scientific goals is to estimate the relationship between brain structure and behavior, especially vocal and tool use behavior. This is complicated by the existence of unobserved age-related developmental confounds and substantial missing data, since (i) for some brains there are none or little *in vivo* data, and (ii) most individuals with behavior samples are (luckily) still alive. To address these problems, we construct structural causal models (Pearl, 1995) and then use these models to (1) simulate synthetic observations, (2) design a data analysis pipeline for estimation and missing data imputation, (3) validate the pipeline using the synthetic data, and (4) analyze the observed sample. For missing data imputation, we use a fully Bayesian network representation (Mohan and Pearl, 2021) and draw samples from the posterior distribution using Hamiltonian Monte Carlo, as described in McElreath, Chapter 15 (McElreath, 2020). The approach is described in detail with working code at https://github.com/rmcelreath/EBC_brain_vocal_modeling. In a later phase, we intend to develop a dynamic causal model of the reciprocal brain and vocal development, using continuous time structural equations as implemented for example in Driver et al. (2017).

## Discussion

The present multidisciplinary methodological approach will provide novel data concerning the evolution of brain structure and its relation to function in non-human primates by capturing variability across individuals, populations, species and ontogeny.

Functional neuroanatomy can be investigated in humans and non-human primates using functional MRI. Extensive neuroimaging research has been conducted on macaques. However, due to ethical constraints comparable data on our closest relatives, chimpanzees and bonobos, cannot be acquired. The presented pipeline here allows linking brain structure to its function by relating anatomical data of high granularity to behavioral data of non-human primates, including animals from diverse socio-ecologies. These structure–function relationships can be inferred for different types of behavior, such as vocal production, tool use or social interaction. It has been argued that the size of certain brain regions changed to some extent in hominoids during evolution, this holds for the frontal cortex (Zilles and Amunts, 2018; Gallardo et al., 2023) as well as for the parietal cortex (Bruner, 2018). Both these cortices support important functions in humans and in non-human primates. The parietal cortex which integrates sensory information from different modalities is known to support diverse mental processes from basic attention to language and social cognition (Numssen et al., 2021), but it is also known to be involved in tool use in humans and in chimpanzees (Hecht et al., 2013). Thus these regions and their connections to other regions within specific neural networks are relevant for different types of behavior that can be observed in non-human and human primates. Here, for brevity, we will focus on one example demonstrating how this methodological pipeline can be applied to chimpanzees and how this can impact discussions on the structure–function relation in hominoid evolution. We illustrate the interplay between brain structure and function focusing on brain structures whose homologs subserve language in the human brain, a key cognitive ability of humans. Nonetheless, our approach is versatile and can be extended to analyze other cognitive functions, such as tool use and social interaction.

The emergence of language and its evolution has been discussed for decades. Neurobiological approaches have tried to identify brain structures that are unique for humans. For example, the work by Rilling et al. (2008) investigating macaque, chimpanzee and human brain connectivity suggested that a particular white matter fiber tract, the arcuate fascicle, running dorsally and connecting the temporal lobe to the inferior frontal cortex differs between these animals - both with respect to its strength and its termination points. This dorsal fiber tract is strongest in the human brain, somewhat weaker in the chimpanzee brain and weakest in the macaque brain. Moreover, it appears that in the human brain the dorsal tract enters more deeply into the middle temporal gyrus compared to the chimpanzee brain. These differences have been interpreted as crucial for the evolution of language capacity which is observed in humans, but not in the chimpanzee. However, there are some limitations impacting this conclusion. First, the data of this study came from only four zoo-living chimpanzees, thereby hampering generalizations at the species level, even though the analysis was later extended to include 29 chimpanzees (Bryant et al., 2020). Second, the dMRI analysis techniques available at that time did not allow for clear differentiation of the termination points of the dorsal fiber tract, neither in the temporal nor in the frontal cortex.

The termination points, however, are of particular importance both in the temporal and the frontal cortex. In the temporal cortex the middle temporal gyrus in humans is thought to support semantic processes most relevant to capture meaning. In the frontal cortex there are three cytoarchitectonically different, but adjacently located areas in the human and the non-human primate brain—which in humans serve different functions. These are area 44 and 45 that in humans are known to be involved in language, and area 6 known to support motor actions including motoric aspects of speech. In human adults the dorsal fiber tract connecting the temporal and frontal cortex has two termination points, one in area 44 and one in area 6 (Perani et al., 2011), with the fiber tract terminating in area 44 subserving the processing of complex sentences (Skeide et al., 2016). In human prelinguistic infants, however, only the fiber tract to area 6 is myelinated whereas the one to area 44 matures much later (Perani et al., 2011; Brauer et al., 2013). This difference between the infant and the adult human brain concerning the termination points of the language-relevant dorsal fiber tract raises the question of the respective structure in our closest relatives. Therefore, it would be of major interest to analyze the target area of the dorsal fiber tract in the chimpanzee brain. Given that these areas differ in their microstructure they can be differentiated histologically also in non-human primates, comparative histological analyses can provide an important contribution for an adequate description of the evolution of the neural basis of language.

The present methodological approach, with its different units as outlined in Figure 1, provides novel details concerning the macro- and microstructure of the neural networks of different cognitive and social capacities and their evolution. Each unit capitalizes on the latest but established methods in their fields, which have been specifically adapted for this methodological pipeline and validated on a separate data set. The present approach goes beyond earlier work by relating ultra-high resolution multi-modal structural data of the brain to rich behavioral data collected in the different individuals *in vivo*. Previous research of the different units and the present examples show that we are able (i) to collect and systematically analyze behavioral data from primates across species, populations and ontogeny (Behavioral Data Collection Unit); (ii) to specify the structure of the fiber tract (Diffusion MRI Unit); (iii) to specify the microstructure and thereby the location of human analog target regions in different cortices (Quantitative MRI Unit); (iv) to specify the histological cytoarchitecture structure of the fiber tracts’ cortical target regions (Histology Unit); (v) to analyze the variations of the endocranial imprints of the skull and relate these to the fossil record (Hominoid Fossil Unit). Additional analyses of data from the different units allows us to (vi) relate behavioral and brain structural aspects not only to behavior, but also to the socio-ecological environment of the individuals (across zoos, sanctuaries and field sites); (vii) to relate brain structural aspects and behavior to development (age of animal); and (viii) to compare all these brain structural aspects across species providing phylogenetic perspective. (viii) Structural causal and Bayesian models (Statistics Unit) optimally leverage the rich dataset to assess variation across species, populations, individuals and across ontogeny. However, the present pipeline requires a longitudinal planning in order to allow to relate *in vivo* behavior to *post-mortem* brain analyses at the macro- and micro-level. Considering that it is unlikely that a complete set of data points will be available for each animal, Bayesian models will help us to impute missing data as described in McElreath (2020).^5^ The resulting data and findings will allow us to elucidate the evolution of the neural basis underlying language, and other behavioral domains providing new answers to the evolution of cognitive and social abilities across species.

The methodological pipeline we present here is unique in that it combines *post mortem* brain structural methods with *in vivo* detailed observational setup, including those under natural conditions. This multi-methodological approach will lead to novel findings concerning the evolution of the brain and its function, undiscovered up to now. Up to now, only results from the units early in the pipeline are published in individual articles (Kopp et al., 2021; Girard-Buttoz et al., 2022a,b; Bortolato et al., 2023a,b; Eichner et al., 2023; Grampp et al., 2023; Gräßle et al., 2023). Results from the other units as well as combined results of the entire pipeline will only appear later due to the logical fact that the time at which behavioral data are gathered from the animals and the point at which their brain are available for brain structure analyses is not predictable and can take years.

Limitations of the present approach remain mainly due to ethical, practical and medical issues. First, due to ethical constraints on gathering functional imaging data, the present approach provides the only possibility to relate brain structure to behavior in great apes. Second, due to the fact that we only include brains of animals that die a natural or unavoidable death, some brains may be lesioned or otherwise pathological. Although in our work we control for this with histopathology, it should be kept in mind when interpreting the data.

The methodological approach presented here could in principle be established elsewhere in a collaboration of different institutions and/or different relevant species that are highly endangered and strongly protected.

The current pipeline will deliver novel results concerning primate brain evolution and will provide a solid basis for developing new hypotheses not only for non-human, but also for human primates which—at least for humans—could be tested using functional imaging. It will enlighten our knowledge of the human brain’s past and, moreover, broaden our knowledge about the human brain’s present.

## Data availability statement

The original contributions presented in the study are included in the article/further inquiries can be directed to the corresponding authors.

## Ethics statement

The animal study was approved by National Research and Animal Welfare Authorities in the Country of Origin, and the Ethics committee of the Max Planck Society for Field Research. The research follows the IUCN best practice guideline for health monitoring and disease control in great ape populations. Brains were extracted postmortem from naturally deceased individuals. In rare cases we also received brains of individuals from European zoos that were euthanized due to terminal medical conditions, or from the wild after human-animal conflict. None of these events were under the control of anybody involved in the research. Since our procedure was non-harmful to the animal, the procedure is considered non-invasive from an ethical point of view. The study was conducted in accordance with the local legislation and institutional requirements.

## Author contributions

AF: Conceptualization, Investigation, Methodology, Supervision, Visualization, Writing – original draft, Writing – review & editing. RW: Conceptualization, Investigation, Methodology, Supervision, Visualization, Writing – original draft, Writing – review & editing. AA: Investigation, Methodology, Visualization, Writing – original draft, Writing – review & editing. CE: Investigation, Methodology, Visualization, Writing – original draft, Writing – review & editing. TG: Investigation, Methodology, Visualization, Writing – original draft, Writing – review & editing. CJ: Investigation, Methodology, Visualization, Writing – original draft, Writing – review & editing. EK: Writing – original draft, Writing – review & editing, Investigation, Methodology, Visualization. IL: Investigation, Methodology, Visualization, Writing – original draft, Writing – review & editing. AD: Methodology, Writing – review & editing. LE: Investigation, Methodology, Visualization, Writing – original draft, Writing – review & editing. CG-B: Investigation, Methodology, Visualization, Writing – original draft, Writing – review & editing. AJ: Investigation, Methodology, Visualization, Writing – original draft, Writing – review & editing. KK: Investigation, Methodology, Visualization, Writing – original draft, Writing – review & editing. MP: Investigation, Methodology, Visualization, Writing – original draft, Writing – review & editing. KP: Investigation, Methodology, Visualization, Writing – original draft, Writing – review & editing. SU: Methodology, Writing – review & editing. DH: Investigation, Methodology, Writing – review & editing. FL: Investigation, Methodology, Visualization, Writing – original draft, Writing – review & editing. RM: Investigation, Methodology, Visualization, Writing – original draft, Writing – review & editing. MM: Investigation, Methodology, Visualization, Writing – original draft, Writing – review & editing. PG: Investigation, Methodology, Visualization, Writing – original draft, Writing – review & editing, Conceptualization. NW: Conceptualization, Investigation, Methodology, Supervision, Visualization, Writing – original draft, Writing – review & editing. CC: Conceptualization, Investigation, Methodology, Supervision, Visualization, Writing – original draft, Writing – review & editing.

## EBC consortium

Daniel Ashoff^14^, Karoline Albig^15^, Bala Amarasekaran^16^, Sam Angedakin^17,18^, Alfred Anwander^1^, Caroline Asiimwe^19^, Christian Bock^20^, Birgit Blazey^21^, Andreas Bernhard^22^, Jacinta C Beehner^23,24^, Laurent Bailanda^25^, Raphael Belais^26^, Thore J Bergman^23,24^, Denny Böttcher^27^, Tatiana Bortolato^2,3,4^, Penelope Carlier^4,5^, Julian Chantrey^28^, Catherine Crockford^2,3,4^, Daniela Denk^29^, Tobias Deschner^30,31^, Ariane Düx^5,8^, Luke J. Edwards^6^, Cornelius Eichner^1^, Dag Encke^32^, Gelardine Escoubas^31^, Malak Ettaj^31^, Pawel Fedurek^19,33^, Karina Flores^16^, Alejandra Romero Florero^16^, Richard Franke^34^, Angela D Friederici^1^, Cedric Girard-Buttoz^2,3,4^, Jorge Gomez Fortun^4,5^, Tobias Gräßle^5^, Eva Gruber-Dujardin^14^, Philipp Gunz^12^, Susan Hambrecht^35^, Florian Hansmann^27^, Jess Hartel^17,36^, Daniel BM Haun^9^, Michael Henshall^37^, Catherine Hobaiter^19,38^, Noémie Hofman^4^, Jennifer E Jaffe^4,5^, Carsten Jäger^6^, Anna Jauch^6^, Stomy Karhemere^39^, Evgenya Kirilina^6^, Robert Klopfleisch^40^, Tobias Knauf-Witzens^41^, Kathrin Kopp^9^, Bastian Lange^42^, Kevin E Langergraber^18,43^, Arne Lawrenz^44^, Kevin Lee^18,43^, Fabian H Leendertz^5,8^, Illona Lipp^6^, Matyas Liptovszky^45^, Christelle Patricia Lumbu^39^, Patrice Makouloutou Nzassi^46^, Guy Landry Mamboundou Kouima^31,46^, Kerstin Mätz-Rensing^14^, Richard McElreath^11^, Zoltan Mezö^47^, Fanny Minesi^26^, Sophie Moittie^45^, Torsten Møller^48^, Markus Morawski^7^, Dave Morgan^49,50^, Mathias Müller^51^, Timothy Mugabe^18,52^, Martin Muller^17,53^, Karin Olofsson-Sannö^54^, Alain Ondzie^55^, Emily Otali^17^, Michael Paquette^1^, Simone Pika^30,31^, Kerrin J. Pine^6^, Andrea Pizarro^16^, Kamilla Pleh^4,5^, Sandra Reichler-Danielowski^56^, Jessica Rendel^44^, Martha M Robbins^57,58^, Konstantin Ruske^59^, Liran Samuni^4,60^, Crickette Sanz^50,61^, Jan Schinköthe^27^, André Schüle^62^, Ingo Schwabe^21^, Katarina Schwalm^21^, Anistan Sebastiampillai^63^, Lara Southern^30,31^, Sheri Speede^64^, Jonas Steiner^4,5^, Mark F Stidworthy^29^, Martin Surbeck^60,65^, Claudia A. Szentiks^46^, Tanguy Tanga^31,46^, Tobias Loubser Theron^37^, Reiner Ulrich^27^, Steve Unwin^10^, Erica van de Waal^37,66^, Sue Walker^67^, Nikolaus Weiskopf^6,13^, Gudrun Wibbelt^47^, Navena Widulin^63^, Hermann Will^68^, Roman M Wittig^2,3,4^, Kim Wood^69^, Emiliano Zaccarella^1^, Klaus Zuberbühler^19,38,70^.

^14^Veterinary Unit, German Primate Center, Göttingen, Germany; ^15^Halle Zoo, Halle / S., Germany; ^16^Tacugama Chimpanzee Sanctuary, Freetown, Sierra Leone; ^17^Kibale Chimpanzee Project, Kibale National Park, Uganda; ^18^Ngogo Chimanzee Project, Kibale National Park, Uganda; ^19^Budongo Conservation Field Station, Masindi, Uganda; ^20^Alfred-Wegener-Institut, Helmholtz-Zentrum für Polar- und Meeresforschung, Bremerhaven, Germany; ^21^Chemisches und Veterinäruntersuchungsamt Stuttgart, Fellbach, Germany; ^22^Zoo Leipzig, Leipzig, Germany; ^23^Department of Psychology, University of Michigan, Ann Arbor, MI, USA; ^24^Capuchins at Taboga Project, Taboga, Costa Rica; ^25^Malebo Bonobo Project, WWF, Kinshasa, DRC; ^26^Lola ya Bonobo, Khinshasa, DRC; ^27^Institute of Veterinary Pathology, Leipzig University, Germany; ^28^Veterinary Pathology and Preclinical Sciences, School of Veterinary Science, Universitiy of Liverpool, UK; ^29^International Zoo Veterinary Group, Keighley, UK; ^30^Comparative BioCognition, Institute of Cognitive Science, Osnabrück University, Germany; ^31^Ozouga Chimpanzee Project, Loango National Park, Gabon; ^32^Zoo Nürnberg, Nürnberg, Germany; ^33^Department of Psychology, University of Stirling, UK; ^34^Zoo Saarbrücken, Saarbrücken, Germany; ^35^Zoo Magdeburg, Magdeburg, Germany; ^36^Department of Biology, Metropolitan Community College, Kansas City, MO; ^37^Inkawu Vervet Project, Mwana Game Reserve, South Africa; ^38^Department of Psychology and Neuroscience, University of St. Andrews; ^39^Institut National de la Recherche Biomédicale, Kinshasa, DRC; ^40^Institute of Veterinary Pathology, Free University Berlin, Germany; ^41^Wilhelma, Stuttgart, Germany; ^42^Zoo am Meer, Bremberhaven, Germany; ^43^Department of Anthropology, Arizona State University, Phoenix, USA; ^44^Zoo Wuppertal, Wuppertal, Germany; ^45^Twycross Zoo, Little Orton, Leicestershire, UK; ^46^Institut de Recherche en Ecologie Tropicale, Libreville, Gabon; ^47^Department of Wildlife Diseases, Leibniz Institute for Zoo and Wildlife Research, Germany; ^48^Kolmarden Wildlife Park, Kolarden, Sweden; ^49^Lincoln Park Zoo, Chicago, USA; ^50^Goualougo Triangle Ape Project, Congo; ^51^Bayerisches Landesamt für Gesundheit und Lebensmittelsicherheit, Erlangen, Germany; ^52^Veterinary Unit, Uganda Wildlife Authority, Kampala, Uganda; ^53^Deaprtment of Anthropology, University of New Mexico, Albuquerque, USA; ^54^National Veterinary Institute, Uppsala, Sweden; ^55^Wildlife Conservation Society DR Congo; ^56^Heidelberg Zoo, Heidelberg, Germany; ^57^Department of Primatology, MPI for Evolutionary Anthropology, Leipzig, Germany; ^58^Loango Gorilla Project, Loango National Park, Gabon; ^59^Magdeburg Zoo, Magdeburg, Germany; ^60^Department of Human Evolutionary Biology, Harvard University, Cambridge, USA; ^61^Department of Anthropology, Washington University, St. Louise, USA; ^62^Zoo Berlin, Berlin, Germany; ^63^Charite, Universitätsmedizin, Berlin, Germany; ^64^Sanaga Yong Chimpanzee Rescue, Mbargue Forest, Cameroon; ^65^Kokolopori Bonobo Research Project, Kokolopori Reserve, DRC; ^66^Department of Ecology and Evolution, University of Lausanne, Switzerland; ^67^Chester Zoo, Chester, UK; ^68^Zoo Nürnberg, Nürnberg, Germany; ^69^Welsh Mountain Zoo, Clauwyn Bay, UK; ^70^Institute of Biology, University of Neuchatel, Switzerland.

## Footnotes

^1^www.chimpanzeebrain.org

^2^cybertracker.org

^3^hMRI.info

^4^volumegraphics.com

^5^https://github.com/rmcelreath/rethinking
